# Global-cognitive health metrics: A novel approach for assessing cognition impairment in adult population

**DOI:** 10.1371/journal.pone.0197691

**Published:** 2018-05-29

**Authors:** Chia-Kuang Tsai, Tung-Wei Kao, Jiunn-Tay Lee, Chung-Ching Wang, Chung-Hsing Chou, Chih-Sung Liang, Fu-Chi Yang, Wei-Liang Chen

**Affiliations:** 1 Department of Neurology, Tri-Service General Hospital, National Defense Medical Center, Taipei, Taiwan; 2 Graduate Institute of Medical Sciences, National Defense Medical Center, Taipei, Taiwan; 3 Division of Family Medicine, Department of Family and Community Medicine, Tri-Service General Hospital and School of Medicine, National Defense Medical Center, Taipei, Taiwan; 4 Division of Geriatric Medicine, Department of Family and Community Medicine, Tri-Service General Hospital and School of Medicine, National Defense Medical Center, Taipei, Taiwan; 5 Department of Psychiatry, Beitou Branch, Tri-Service General Hospital, National Defense Medical Center, Taipei, Taiwan, Republic of China; University Of São Paulo, BRAZIL

## Abstract

Dementia is the supreme worldwide burden for welfare and the health care system in the 21st century. The early identification and control of the modifiable risk factors of dementia are important. Global-cognitive health (GCH) metrics, encompassing controllable cardiovascular health (CVH) and non-CVH risk factors of dementia, is a newly developed approach to assess the risk of cognitive impairment. The components of ideal GCH metrics includes better education, non-obesity, normal blood pressure, no smoking, no depression, ideal physical activity, good social integration, normal glycated hemoglobin (HbA1c), and normal hearing. This study focuses on the association between ideal GCH metrics and the cognitive function in young adults by investigating the Third Health and Nutrition Examination Survey (NHANES III) database, which has not been reported previously. A total of 1243 participants aged 17 to 39 years were recruited in this study. Cognitive functioning was evaluated by the simple reaction time test (SRTT), symbol-digit substitution test (SDST), and serial digit learning test (SDLT). Participants with significantly higher scores of GCH metrics had better cognitive performance (p for trend <0.01 in three cognitive tests). Moreover, better education, ideal physical activity, good social integration and normal glycated hemoglobin were the optimistic components of ideal GCH metrics associated with better cognitive performance after adjusting for covariates (p < 0.05 in three cognitive tests). These findings emphasize the importance of a preventive strategy for modifiable dementia risk factors to enhance cognitive functioning during adulthood.

## Introduction

Dementia is the supreme worldwide burden for welfare and the health care system in the 21st century. The estimated number of people with dementia will increase from 47 million in 2015 to more than 140 million in 2050 [1]. As dementia deteriorates in the patients, the care costs increase to accommodate the need of life dependence, including daily activity assistance and medical care. The estimated global cost of dementia was approximately 818 billion US dollars in 2015, which will keep increasing because of the increased number of people with dementia in the future [2]. Therefore, the early identification and control of the modifiable risk factors of dementia are important.

Previous studies had linked various independent risk factors with cognitive decline. Cardiovascular health (CVH) factors, including stroke, elevated blood sugar, hypertension, hypercholesterolemia and obesity, are wildly recognized as imperative hazard factors for cognitive function [3,4]. People with a higher number of ideal CVH metrics have a lower risk of dementia. We had reported a similar result that cognitive functioning is negatively correlated with the increased number of components of metabolic syndrome [5].

On the other hand, several non-CVH factors are additionally regarded as risk factors of cognitive decline, including social isolation [6], hearing loss [7], oral health [8], and less education [9]. However, most participants of these studies are older adults. Recently, Livingston et al. [10] proposed combining the global-cognitive health (GCH) metrics with potentially controllable CVH and non-CVH risk factors of dementia. The ideal GCH metrics are composed of nine amendable factors, including education, obesity, blood pressure, smoking, depression, physical activity, social integration, glycated hemoglobin (HbA1c), and normal hearing [10]. Ideal GCH metrics emphasizes that early intervention of these amendable dementia-associated risk factors is potentially beneficial for cognitive reserve [10]. A previous study had investigated the cognitive performance and ideal cardiovascular health in young adults, but they did not incorporate the hearing and social integration [11]. This study focuses on the association between ideal GCH metrics and cognitive function in young adults, which has not been reported previously.

The National Health and Nutrition Examination Survey (NHANES), including a demonstrative sample of the non-institutionalized civilian United States population, is a public database on the website. We investigated datasets from the Third NHANES (NHANES III), ranging from 1988 through 1994, to explore the relationships between the ideal GCH metrics and cognitive function as measured by 3 neurobehavioral tests included in the survey. We hope that this ideal GCH metrics study contributes to the constructive strategy for cognitive reserve in adulthood.

## Materials and methods

### Study design, ethics statement, and study subjects

This is a cross-sectional study, and all data are from the publicly accessible NHANES III database (https://www.cdc.gov/nchs/Tutorials/nhanes/Preparing/Download/Intro_III.htm). The NHANES III study protocol was certified by the Institutional Review Board (IRB) of the National Center for Health Statistics (NCHS). Before participating in the survey for collection procedures and examinations, documented consents were obtained from all eligible participants.

**Fig 1:**
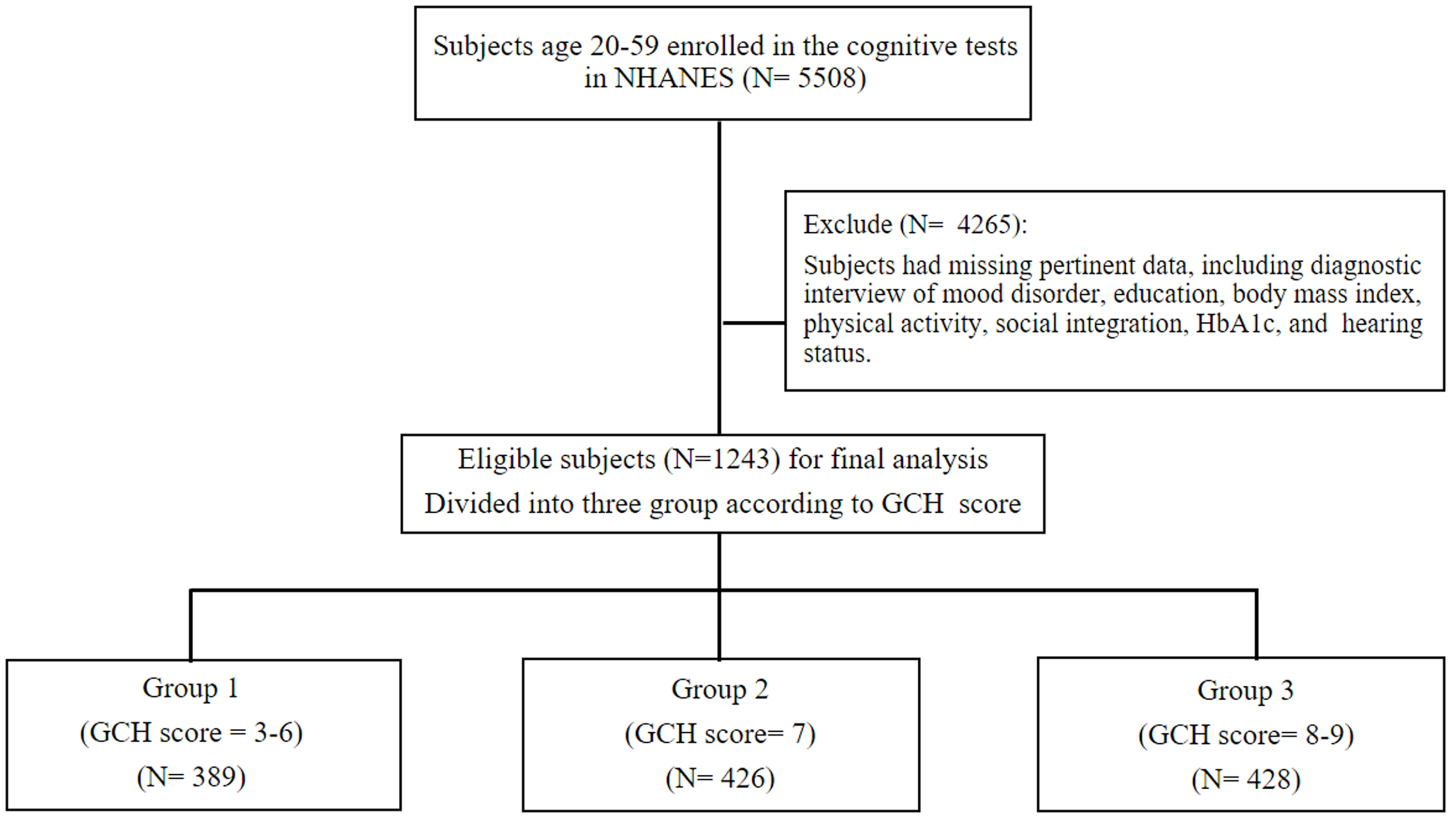
Flowchart showing selection of study subjects for the study.

The Centers for Disease Control and Prevention (CDC) and the NCHS executed NHANES III from 1988 through 1994. The participants were civilian, non-institutionalized individuals representing the US population. Comprehensive information was selected by trained examiners during the home interview, including demographic characteristics, questionnaires, and medical past histories. Our exclusion criteria for participants included missing values regarding education status, body mass index (BMI), blood pressure, smoking, depression, physical activity, social integration, HbA1c, and hearing questionnaires. There were 5508 adults aged 20–59 years taking the neurobehavioral tests (Fig 1). The subjects recruited in this study were aged from 20 to 39 because participants aged > 39 did not undergo an interview for the mood disorder survey. Detailed introductions and protocols of the NHANES III survey have been published [12].

### Definition of ideal GCH metrics

According to the Livingston et al. [10] study, we constructed the GCH metrics as having nine modifiable components, including education, obesity, blood pressure, smoking, depression, physical activity, social integration, HbA1c, and normal hearing. Participants having an education higher than high school were defined as the favorable category. Non-obesity was considered as BMI < 30 kg/m^2^ according to the definition of the World Health Organization. BMI was computed as a person’s body weight in kilograms divided by the square of the person’s height in meters (kg/m^2^). The ideal systolic blood pressure was considered as < 140 mmHg, and the ideal diastolic blood pressure was defined as < 90 mmHg [13]. Non-smoking status includes “never” and “former” smoking based on the American Heart Association (AHA) definition [13].

In NHANES III, only young adults (aged 17 to 39) underwent the Diagnostic Interview Schedule that helps to identify individuals who fulfill the diagnostic criteria of mood disorders based on the DSM-III version [14]. Depression was defined as having a major depressive episode (MDE), and we excluded bereavement-related MDE [15].

The intensity of physical activity was evaluated by the levels of metabolic equivalent tasks (METs) [16–18]. One MET represents the ratio of the energy expenditure of the activity to the resting metabolic rate [16,17]. We categorized the ideal physical activity as engaging in any vigorous activity with > 3 to 5.9 METs five or more times per week or vigorous activity with > 6 METs three or more times per week [18].

We applied a modified social network index (SNI) to access social integration [19]. The SNI has been used in a previous investigation of the NHANES III database [20]. The SNI encompass four fields, and we scored one point for each of the following: (1) current in marital status, (2) more than 156 contacts with close friends and relatives in the past one year, (3) ≥ 4 church or religious activities in the past one year, and (4) participating in community groups. The total SNI score ranged from 0–4. Total scores of 2, 3, and 4 indicated good social integration, while total scores of 0 and 1 indicated social isolation (unfavorable).

HbA1c values < 5.7% were classified as the ideal category according to the suggestions of the American Diabetes Association [21]. Normal hearing is defined as not using a hearing aid.

We constructed a global-cognitive health metrics score (number of global-cognitive health metrics) by recoding the 9 metrics. Each ideal GCH metric was scored as one point.

### Cognitive function testing

In the NHANES III, the participants’ central nervous system function was evaluated by three computerized neurobehavioral tests. During the simple reaction time test (SRTT), the participants pressed a button as quickly as possible whenever a visual or auditory stimulus appeared. Each participant had a total of 50 tests. The participants’ mean reaction time was estimated in milliseconds (ms) [22,23]. The outlier values of reaction time (≤ 50 ms or ≥ 750 ms) were dismissed.

During the symbol-digit substitution test (SDST), the participants were requested to speedily tally the character with the exact parallel digit within 2 minutes. Each participant had a trial with a dissimilar coupling of digits and characters for 4 times. The SDST is frequently applied to assess frontal-lobe-associated functions, including sustained attention, visuospatial dexterity, and speed of motor processing. The score of the SDST was recorded as the average overall time in which the participants accomplished the four tests [5,22,23].

During the serial digit learning test (SDLT), the individuals were asked to remember a series of digits that were shown on a computer screen. The participant only saw one digit displayed at a time. Each digit presented for 6 seconds, with a 6-second break between the digits. The individuals were asked to use the numeric buttons on the keyboard to key in the whole sequence in the order in which they were shown after all digits were displayed. Testing ceased when the individuals responded accurately on two uninterrupted tests or after testing for eight times. The SDLT score was calculated as the summation of the faults encountered for each trial [22,23].

### Covariates

The individuals’ relevant data were partially saved by a computer-assisted personal interviewing system. Demographic data, containing age, sex, race/ethnicity, years of educational, and medical history, were collected. Race/ethnicity was grouped as the following categories: non-Hispanic white, non-Hispanic black, Mexican American, or other. Status of smoking was determined by a questionnaire “Do you now smoke cigarettes?” Self-reported comorbidities including history of congestive heart failure (yes/no), history of stroke (yes/no), and history of chronic bronchitis (yes/no) were collected. The other biochemical covariates, including serum glucose, C-reactive protein (CRP), serum folate, and serum cotinine, were determined using standardized methods with respect to the CDC’s guidelines. Detailed information on specimen collection is openly available on the NHANES website.

### Statistical analyses

We utilized SPSS (Version 18.0 for Windows, SPSS, Inc., Chicago, IL, USA) to investigate the NHANES III data. We tested differences in the characteristics among the GCH groups using analysis of variance and the Chi-Square test. When these computerized cognitive tests were regarded as continuous variables, multivariate linear regression analysis was used between the GCH and cognitive functions. Based on quartile-based analysis, we separated cognitive performances into quartiles, and the reference group was the subjects in the lowest quartile. Next, multivariate logistic regression analyses were conducted to determine the association between the scores and components of GCH and cognition after adjustment for pertinent confounding variables. Unadjusted and adjusted models were applied for adjusting covariates: Model 1 = unadjusted; Model 2 = age, gender, and race/ethnicity; Model 3 = Model 2 + CRP, serum folate, and serum cotinine. Model 4 = Model 3 + past medical histories. Statistical significance was defined as a two-sided p < 0.05.

## Results

### Characteristics of the study subjects

Table 1 demonstrates the demographic and baseline characteristics of the GCH metrics. We divided the 1243 participants into three groups by scores of GCH metrics (group 1: GCH scores 3 to 6; group 2: GCH score 7; group 3: GCH scores 8 and 9). At baseline, there were 389 (31.3%) study participants in group 1, 426 (34.3%) in group 2, and 428 (34.4%) in group 3. The average age at interviews was 30.0 ± 5.5 years old. The group with higher GCH scores exhibited significantly lower serum CRP, systolic and diastolic pressure, BMI, and serum glucose than the group with lower GCH scores (p < 0.05).

**Table 1:** Demographic and baseline characteristics of global-cognitive health metrics.

Number of ideal GCH metrics					
	Total	Group 1 (GCH score = 3–6)	Group 2(GCH score = 7)	Group 3(GCH score = 8–9)	*P* value
*n*	1243	389	426	428	
Continuous Variables, mean (SD)					
Cognitive test					
SRTT	237.5(51.8)	246.06 (53.5)	235.0 (52.6)	232.3 (48.4)	0.001
SDST	2.71 (0.7)	2.94 (0.9)	2.65 (0.8)	2.55 (0.5)	<0.001
SDLT	5.2 (4.5)	6.20 (4.8)	5.22 (4.4)	4.41 (4.2)	<0.001
Age	30.0(5.5)	30.26 (5.6)	29.41 (5.5)	30.45 (5.5)	0.024
Serum glucose (mg/dL)	90.4 (18.3)	92.74 (20.7)	90.05 (20.3)	88.66 (13.1)	0.010
Serum C-reactive protein (mg/dL)	0.3 (0.585)	0.48 (0.7)	0.34 (0.3)	0.34 (0.6)	0.002
Systolic blood pressure (mmHg)	114.6 (12.6)	116.46 (13.9)	114.16 (12.7)	113.61 (11.2)	<0.001
Diastolic blood pressure (mmHg)	70.7 (12.9)	72.43 (14.3)	70.66 (11.9)	69.32 (12.4)	0.001
Serum folate (ng/mL)	4.9 (3.4)	4.32 (2.7)	4.89 (3.2)	5.64 (4.1)	<0.001
Body mass index	26.1 (5.6)	28.35 (6.8)	25.55 (5.4)	24.80 (3.9)	<0.001
Serum cotinine (ng/mL)	154.5(161.5)	173.19 (155.5)	183.90 (168.2)	108.52 (149.8)	<0.001
Categorical variables, n (%)					
Male, *n* (%)	663 (53.3)	190 (48.8)	239 (56.1)	234 (54.7)	0.138
Race/ethnicity					
Non-Hispanic white	473 (38.1)	102 (26.2)	159 (37.3)	212 (49.5)	<0.001
Congestive heart failure	8 (0.6)	4 (1.0)	4 (0.9)	0 (0)	0.215
Stroke	2 (0.2)	2 (0.5)	0 (0)	0 (0)	0.54
Chronic bronchitis	72 (5.8)	27 (6.9)	27 (6.3)	18 (4.2)	0.095
Ideal GCH metrics					
Education (>high school)	1010 (81.3)	231 (59.4)	372 (87.3)	407 (95.1)	<0.001
Non-obesity (BMI < 30)	995 (88.0)	244 (62.7)	357 (83.8)	394 (92.1)	<0.001
Normal Blood pressure (<140/90 mmHg)	1180 (94.9)	344 (88.4)	411 (96.5)	425 (99.3)	<0.001
No smoking	874 (70.3)	44 (11.3)	342 (80.3)	187 (43.7)	<0.001
No depression	1094 (88.0)	300 (77.1)	375 (88.0)	419 (97.9)	<0.001
Ideal physical activity	1039 (83.6)	237 (60.9)	380 (89.2)	422 (98.6)	<0.001
Good social integration (SNI = 2–4)	634 (51.0)	85 (21.9)	187 (43.9)	362 (84.6)	<0.001
Normal glycated hemoglobin (< 5.7%)	1112 (89.5)	298 (76.6)	391 (91.8)	423 (98.8)	<0.001
Normal hearing (%)	1240 (99.8)	388 (99.7)	425 (99.8)	427 (99.8)	0.908

BMI, body mass index; SNI, social network index; SD, standard deviation; SDLT, Serial Digit Learning Test; SDST, Symbol Digit Substitution Test; SRTT, Simple Reaction Time Test; BP, blood pressure.

Table 2 presents multiple logistic regression analysis of associations between the number of GCH metrics and cognitive function. After adjusting all covariates in model 4, these associations were all statistically significant, as the presence of each component of the ideal GCH metrics predicted a better performance of SDLT, SDST, and SRTT (higher SDLT, SDST, and SRTT scores are associated with worse performance for each of these tests) (p < 0.01).

**Table 2:** Associations between number of ideal GCH metrics and cognitive function.

Variables	Model 1		Model 2		Model 3		Model 4	
	β (95% CI)	P-value	β (95% CI)	P-value	β (95% CI)	P-value	β (95% CI)	P-value
	SDLT							
Number of GCH metrics	-0.703 (-0.921, -0.485)	<0.001	-0.507 (-0.716, -0.297)	<0.001	-0.437 (-0.655, -0.219)	<0.001	-0.429 (-0.648, -0.210)	<0.001
	SRTT							
Number of GCH metrics	-5.392 (-7.899, -2.885)	<0.001	-4.180 (-6.692, -1.668)	0.001	-4.316 (-6.932, -1.700)	0.001	-4.210 (-6.835, -1.585)	0.002
	SDST							
Number of GCH metrics	-0.141 (-0.178, -0.104)	<0.001	-0.115 (-0.151, -0.080)	<0.001	-0.101 (-0.138, -0.064)	<0.001	-0.101 (-0.138, -0.063)	<0.001

CI, confidence interval; GCH, global-cognitive health; SDLT, Serial Digit Learning Test; SDST, Symbol Digit Substitution Test; SRTT, Simple Reaction Time Test;

Model 1 unadjusted;

Model 2 adjusted for sex, race-ethnicity and age.

Model 3 adjusted for model 2 plus C-reactive protein, serum folate, and serum cotinine.

Model 4 adjusted for model 3 plus congestive heart failure, stroke, and chronic bronchitis.

In Table 3, we further compared the association between subgroups with different numbers of ideal GCH metrics and cognitive functioning. Compared with Group 1 individuals with 3 to 6 ideal GCH metrics, Group 3 with more than 7 ideal GCH metrics demonstrated better cognitive performance in the three cognitive tests with statistical significance (p < 0.05).

**Table 3:** Association between groups of GCH and cognitive function (as continuous variables).

Variables	Model 1		Model 2		Model 3		Model 4	
	β (95% CI)	P-value	β (95% CI)	P-value	β (95% CI)	P-value	β (95% CI)	P-value
Groups of GCH metrics	SDLT							
G2 vs G1	-0.998 (-1.628, -0.369)	0.002	-0.652 (-1.251, -0.053)	0.033	-0.589 (-1.191, 0.013)	0.055	-0.566 (-1.168, 0.037)	0.066
G3 vs G1	-1.835 (-2.463, -1.206)	<0.001	-1.343 (-1.946, -0.741)	<0.001	-1.144 (-1.766, -0.521)	<0.001	-1.111 (-1.736, -0.487)	0.001
P for trend	<0.001		<0.001		<0.001		<0.001	
Groups of GCH metrics	SRTT							
G2 vs G1	-10.469 (-17.698, -3.240)	0.005	-7.472 (-14.641, -0.304)	0.041	-7.291 (-14.514, -0.067)	0.048	-7.031 (-14.262, 0.200)	0.057
G3 vs G1	-13.534 (-20.738, -6.330)	<0.001	-10.005 (-17.201, -2.808)	0.006	-10.115 (-17.572, -2.659)	0.008	-9.741 (-17.220, -2.261)	0.011
P for trend	<0.001		0.003		0.004		0.006	
Groups of GCH metrics	SDST							
G2 vs G1	-0.281 (-0.387, -0.174)	<0.001	-0.236 (-0.339, 0.134)	<0.001	-0.232 (-0.335, 0.129)	<0.001	-0.233 (-0.336, 0.130)	<0.001
G3 vs G1	-0.391 (-0.497, -0.284)	<0.001	-0.328 (-0.431, -0.225)	<0.001	-0.285 (-0.392, -0.179)	<0.001	-0.285 (-0.391, -0.178)	<0.001
P for trend	<0.001		<0.001		<0.001		<0.001	

CI, confidence interval; GCH, global-cognitive health; SDLT, Serial Digit Learning Test; SDST, Symbol Digit Substitution Test; SRTT, Simple Reaction Time Test;

Model 1 unadjusted;

Model 2 adjusted for sex, race-ethnicity and age.

Model 3 adjusted for model 2 plus C-reactive protein, serum folate, and serum cotinine.

Model 4 adjusted for model 3 plus congestive heart failure, stroke, and chronic bronchitis.

In Table 4, we use group 1 with 3–6 components of GCH metrics as the baseline; the proportion of poor cognitive performance was significantly decreased in the highest GCH score group compared with the lowest GCH score group (SDLT: OR 0.542, 95% CI 0.367–0.800, p = 0.002; SRTT: OR 0.569, 95% CI 0.397–0.815, p = 0.002; SDST: OR 0.420, 95% CI 0.277–0.636, p < 0.001) (model 4).

**Table 4:** Association between groups of GCH and poor cognitive function (as categorical variables).

Variables	Model 1		Model 2		Model 3		Model 4	
	OR (95% CI)	P-value	OR (95% CI)	P-value	OR (95% CI)	P-value	OR (95% CI)	P-value
Groups of GCH metrics	Poor cognition (lowest quartile of SDLT)							
G2 vs G1	0.612 (0.437, 0.857)	0.004	0.706 (0.496, 1.005)	0.053	0.734 (0.515, 1.048)	0.089	0.750 (0.525, 1.073)	0.115
G3 vs G1	0.418 (0.291, 0.601)	<0.001	0.482 (0.330, 0.702)	<0.001	0.529 (0.359, 0.779)	0.001	0.542 (0.367, 0.800)	0.002
Groups of GCH metrics	Poor cognition (lowest quartile of SRTT)							
G2 vs G1	0.572 (0.411, 0.795)	0.001	0.629 (0.450, 0.880)	0.007	0.644 (0.459, 0.902)	0.011	0.654 (0.466, 0.918)	0.014
G3 vs G1	0.494 (0.353, 0.691)	<0.001	0.551 (0.390, 0.778)	0.001	0.554 (0.388, 0.793)	0.001	0.569 (0.397, 0.815)	0.002
Groups of GCH metrics	Poor cognition (lowest quartile of SDST)							
G2 vs G1	0.508 (0.357, 0.724)	<0.001	0.551 (0.379, 0.800)	0.002	0.563 (0.386, 0.821)	0.003	0.559 (0.383, 0.817)	0.003
G3 vs G1	0.348 (0.237, 0.511)	<0.001	0.386 (0.258, 0.578)	<0.001	0.416 (0.275, 0.629)	<0.001	0.420 (0.277, 0.636)	<0.001

CI, confidence interval; GCH, global-cognitive health; SDLT, Serial Digit Learning Test; SDST, Symbol Digit Substitution Test; SRTT, Simple Reaction Time Test;

Model 1 unadjusted;

Model 2 adjusted for sex, race-ethnicity and age.

Model 3 adjusted for model 2 plus C-reactive protein, serum folate, and serum cotinine.

Model 4 adjusted for model 3 plus congestive heart failure, stroke, and chronic bronchitis.

### Regression coefficients of components of GCH metrics for the SDLT, SRTT, and SDST

The results of the applications of the models that tested the effects of each component of ideal GCH metrics on the SDLT, SRTT, and SDST are illustrated in Tables 5–7. There are four GCH components, including education higher than high school, ideal physical activity, HbA1c < 5.7, and social integration, that significantly and negatively correlate with the SDLT, SRTT, and SDST scores in the fully adjusted models (p<0.01). Furthermore, education higher than high school was the most influential variable in reducing the association magnitudes, and ideal physical activity was the second most significant feature that was associated with cognitive performance.

**Table 5:** Regression coefficients of components of global-cognitive health for SDLT.

Variables	Model 1		Model 2		Model 3		Model 4	
	β (95% CI)	P-value	β (95% CI)	P-value	β (95% CI)	P-value	β (95% CI)	P-value
Components of GCH metrics								
Better education	-0.383 (-5.287, -4.596)	<0.001	-0.296 (-4.170, -3.474)	<0.001	-0.290 (-4.089, -3.394)	<0.001	-0.289 (-4.076, -3.380)	<0.001
Non-obesity	-0.088 (-1.333, -0.673)	<0.001	-0.030 (-0.659, -0.034)	0.030	-0.024 (-0.594, 0.044)	0.091	-0.023 (-0.586, 0.052)	0.101
Normal Blood pressure	-0.100 (-2.111, -1.169)	<0.001	-0.051 (-1.280, -0.374)	<0.001	-0.045 (-1.192, -0.289)	0.001	-0.044 (-1.168, -0.265)	0.002
No smoking	-0.032 (-0.746, 0.095)	0.129	-0.088 (-1.296, -0.497)	<0.001	-0.013 (-0.646, 0.377)	.606	-0.011 (-0.628, 0.394)	0.654
No depression	0.029 (-0.137, 1.020)	0.135	0.023 (-0.198, 0.903)	0.209	0.025 (-0.171, 0.929)	0.176	0.025 (-0.159, 0.944)	0.163
Ideal physical activity	-0.163 (-2.534, -1.780)	<0.001	-0.107 (-1.771, -1.056)	<0.001	-0.100 (-1.673, -0.958)	<0.001	-0.098 (-1.647, -0.932)	<0.001
Good social integration	-0.036 (-0.663, -0.071)	0.015	-0.048 (-0.765, -0.215)	<0.001	-0.037 (-0.657, -0.105)	0.007	-0.037 (-0.654, -0.102	0.007
Normal glycated hemoglobin	-0.156 (-2.546, -1.755)	<0.001	-0.075 (-1.419, -.650)	<0.001	-0.062 (-1.243, -.466)	<0.001	-0.061 (-1.232, -.455)	<0.001
Normal hearing	-0.020 (-3.564, 0.661)	0.178	-0.006 (-2.384, 1.527)	0.668	-0.008 (-2.496, 1.395)	0.579	-0.008 (-2.514, 1.376)	0.566

CI, confidence interval; GCH, global-cognitive health; SDLT, Serial Digit Learning Test; SDST, Symbol Digit Substitution Test; SRTT, Simple Reaction Time Test;

Model 1 unadjusted;

Model 2 adjusted for sex, race-ethnicity and age.

Model 3 adjusted for model 2 plus C-reactive protein, serum folate, and serum cotinine.

Model 4 adjusted for model 3 plus congestive heart failure, stroke, and chronic bronchitis.

**Table 6:** Regression coefficients of components of global-cognitive health for SRTT.

Variables	Model 1		Model 2		Model 3		Model 4	
	β (95% CI)	P-value	β (95% CI)	P-value	β (95% CI)	P-value	β (95% CI)	P-value
Components of GCH metrics								
Better education	-0.172 (-27.945, -20.064)	<0.001	-0.155(-25.722, -17.539)	<0.001	-0.151 (-25.187, -16.991)	<0.001	-0.150 (-25.018, -16.822)	<0.001
Non-obesity	-0.074 (-12.979, -5.754)	<0.001	-0.036 (-8.126, -0.910)	0.014	-0.036 (-8.253, -0.849)	0.016	-0.035 (-8.112, -0.711)	0.019
Normal Blood pressure	-0.021 (-8.860, 1.451)	0.159	-0.020 (-8.805, 1.637)	0.178	-0.017 (-8.305, 2.141)	0.247	-0.015 (-7.997, 2.446)	0.297
No smoking	-0.051 (-10.100, -1.105)	0.015	-0.060 (-11.159, -2.126)	0.004	-0.017 (-7.672, 3.906)	0.524	-0.014 (-7.368, 4.194)	0.590
No depression	0.017 (-3.514, 9.649)	0.361	0.041(0.722, 13.613)	0.029	0.041 (0.879, 13.775)	0.026	0.042 (1.020, 13.957)	0.023
Ideal physical activity	-0.137 (-24.005, -15.802)	<0.001	-0.100 (-18.592, -10.381)	<0.001	-0.096 (-17.986, -9.764)	<0.001	-0.094 (-17.679, -9.450)	<0.001
Good social integration	-0.037 (-7.366, -0.908)	0.012	-0.046(-8.412, -2.074)	0.001	-0.040 (-7.664, -1.279)	0.006	-0.039 (-7.603, -1.220)	0.007
Normal glycated hemoglobin	-0.068 (-14.662, -5.982)	<0.001	-0.055 (-12.863, -4.008)	<0.001	-0.049 (-12.012, -3.039)	0.001	-0.049 (-11.917, -2.948)	0.001
Normal hearing	-.004 (-26.851, 19.986)	0.774	-0.001 (-24.118, 21.681)	0.917	-0.003 (-25.195, 20.548)	0.842	-0.003 (-25.571, 20.139)	0.816

CI, confidence interval; GCH, global-cognitive health; SDLT, Serial Digit Learning Test; SDST, Symbol Digit Substitution Test; SRTT, Simple Reaction Time Test;

Model 1 unadjusted;

Model 2 adjusted for sex, race-ethnicity and age.

Model 3 adjusted for model 2 plus C-reactive protein, serum folate, and serum cotinine.

Model 4 adjusted for model 3 plus congestive heart failure, stroke, and chronic bronchitis.

**Table 7:** Regression coefficients of components of global-cognitive health for SDST.

Variables	Model 1		Model 2		Model 3		Model 4	
	β (95% CI)	P-value	β (95% CI)	P-value	β (95% CI)	P-value	β (95% CI)	P-value
*Components of GCH metrics*								
Better education	-0.398 (-1.276, -1.118)	<0.001	-0.333 (-1.081, -0.924)	<0.001	-0.325 (-1.055, -0.899)	<0.001	-0.323 (-1.050, -0.894)	<0.001
Non-obesity	-0.077 (-0.293, -0.135)	<0.001	-0.019 (-0.127, 0.022)	0.170	-0.015 (-0.117, 0.035)	0.288	-0.014 (-0.114, 0.037)	0.318
Normal Blood pressure	-0.115 (-0.567, -0.343)	<0.001	-0.030 (-0.228, -0.012)	0.029	-0.024 (-0.200, 0.013)	0.086	-0.022 (-0.192, 0.021)	0.118
No smoking	-0.035 (-0.188, 0.013)	0.089	-0.119 (-0.389, -0.200)	<0.001	-0.016 (-0.159, 0.081)	0.528	-0.014 (-0.154, 0.086)	0.581
No depression	0.038 (0.002, 0.202)	0.045	0.024 (-0.031, 0.159)	0.188	0.027 (-0.023, 0.166)	0.139	0.028 (-0.020, 0.170)	0.121
Ideal physical activity	-0.160 (-0.598, -0.419)	<0.001	-0.121 (-0.467, -0.298)	<0.001	-0.111 (-0.437, -0.269)	<0.001	-0.108 (-0.426, -0.258)	<0.001
Good social integration	-0.060 (-0.219, -0.077)	<0.001	-0.083 (-0.272, -0.141)	<0.001	-0.068 (-0.234, -0.103)	<0.001	-0.068 (-0.234, -0.104)	<0.001
Normal glycated hemoglobin	-0.191(-0.730, -0.543)	<0.001	-0.087 (-0.382, -0.200)	<0.001	-0.071(-0.328, -0.145)	<0.001	-0.069 (-0.322, -0.139)	<0.001
Normal hearing	-0.018 (-0.843, 0.182)	0.206	0.003 (-0.411,0.533)	0.799	0.001 (-0.445, 0.489)	0.927	0.001 (-0.448, 0.485)	0.937

CI, confidence interval; GCH, global-cognitive health; SDLT, Serial Digit Learning Test; SDST, Symbol Digit Substitution Test; SRTT, Simple Reaction Time Test;

Model 1 unadjusted;

Model 2 adjusted for sex, race-ethnicity and age.

Model 3 adjusted for model 2 plus C-reactive protein, serum folate, and serum cotinine.

Model 4 adjusted for model 3 plus congestive heart failure, stroke, and chronic bronchitis.

## Discussion

This cross-sectional study based on the NHANES III study proposed that there was a positive correlation between the number of ideal GCH metrics and better cognitive performance in adulthood. After adjusting for sex, race-ethnicity, age, CRP, serum folate and cotinine, and congestive heart failure, stroke, and chronic bronchitis, participants with more than 7 components of GCH metrics had a lower risk of poor cognitive functioning. Until now, this is the first study to explore the relationship between the GCH metrics and cognitive performance in young adults.

Early cognitive enrichment had been reported as a protective factor for cognitive deterioration and eventual conversion to dementia later in life. Individuals with lower education attainment are associated with decreased intracranial volume and smaller head size [24]. The cognitive reserve theory was proposed to explain this phenomenon, and years of education is a frequently used substitution for cognitive reserve [25]. Increased cognitive reserve in individuals indicates that they could utilize substitute brain domain or cognitive networks effectively to endure pathological characteristics of Alzheimer’s Disease (AD) as well as aging [25]. Comparing the uptake of Pittsburgh Compound B in individuals with similar cognitive impairment, those with higher years of education demonstrated higher uptake of these amyloid ligands in the brain than those with lower years of education [26]. A similar result was obtained in postmortem studies that high-education persons had higher AD pathological burden than cognitively comparable low-education persons [27]. Moreover, high education attainment is associated with decreasing AD-related cerebrospinal fluid (CSF) biomarkers [28]. However, the participants in the above studies are old adults with an average age of more than 60 years old.

In this study, we observed that education attainment has the most influence on cognitive performance in these young adults. Given that the average age of the participants was approximately 30 years old, it is unlikely that brain atrophy and AD-related pathological burden explain the association between education and cognitive performance. It is more likely that higher education or early cognitive enrichment may contribute to augmented neuronal connectivity or utilize cognitive networks more effectively. The adult hippocampal neurogenesis was first described in rodents living in an enriched habitat [29]. Besides, increased hippocampal-associated stimulations could boost hippocampal neurogenesis [30]. Based on these animal studies, the effect of education-related cognitive protection has been proposed to result from hippocampal neurogenesis. Various degrees of education attainment might result in various levels of cognitive enrichment in humans [31]. A study recruiting 146 healthy participants between the ages of 20–79 demonstrated that high education ameliorates performance of neuropsychological tests [32]. Piras et al. [33] provided a piece of neuroimage-based evidence that education is associated with the deep gray matter changes in the bilateral hippocampus. Moreover, higher education young individuals having medical temporal activity performed better in cognitive tasks, compared with lower education individuals [34]. Therefore, level of education is associated with activation of different brain activity during memory tasks.

These observations emphasize that formal education contributes to improved cognitive performance.

Vigorous activity is the second most influential ideal GCH metric in this study. There is evidence that each session of exercise could promote brain-derived neurotrophic factor (BDNF) activity and that regular exercise could boost BDNF upregulation [35]. In animal studies, rats with exercise training demonstrated improved cognition and synaptic plasticity; however, this exercise-related beneficial effect could be abolished by injection of BDNF blocker in the hippocampus [36–38]. Increased hippocampal BDNF expression by exercise or histone deacetylase (HDAC) inhibitor helps diminish the threshold of successful learning of object location memory [39]. BDNF additionally decreases oxidative-stress-related DNA damage to cortical neurons via upregulating the DNA repair enzyme APE1 [40]. Moreover, exercise increases cerebral blood volume and angiogenesis in the medial temporal memory-related area, such as the dentate gyrus, entorhinal cortex and hippocampus, and has been linked with exercise-provoked neurogenesis in mice [41,42].

In human studies, cardiorespiratory fitness is positively associated with improved cognitive performance from children to adults [43–46]. People with exercise training exhibited selectively increased cerebral blood volume in the dentate gyrus, which is significantly associated with aerobic fitness and cognitive performance [42]. The exercised-induced positive cognitive impact has been additionally reported to correlate with increased hippocampal volume measured by MRI in young and old adults [47,48]. The other beneficial effect of cardiorespiratory fitness includes a decrease in CRP values [49] and an increase in brain metabolism [50] and neurotropic factor BDNF levels [35]. Furthermore, individuals with genetic risk factors for AD exhibit lower β-amyloid 42 and elevated total tau in the CSF that triggers neuron degeneration. However, higher physical activity could diminish the destructive impact of genetic susceptibility on these CSF biomarkers of AD [51].

Hearing impairment, a relatively novel risk factor of dementia in elder people, has been identified in recent years [7]. Hearing impairment may be detrimental to cognitive reserve through mechanisms such as increased mental stress, decreased social network engagement, and boosted brain atrophy [52,53]. Therefore, we incorporated hearing impairment as a component of GCH metrics and aimed to determine whether hearing impairment is an additional important risk factor of cognitive functioning in young adults. However, this study demonstrated no statistical significance between hearing impairment and cognitive performance. Two reasons may explain this negative result. First, the sample size of hearing impairment in this study is too small. Second, the destructive effect of hearing impairment may take longer time to cause cognitive decline because the mean age in previous research is generally over 50 years [54].

Several restrictions exist in our study. First, the NHANES provides cross-sectional, not longitudinal, data. Therefore, we could not evaluate the lasting effect of GCH metrics on the cognitive function of the participants. Additionally, we could not exclude the probability that the components of GCH metrics, such as education attainment, act as confounding factors and cause inverse causality. A well-designed prospective study would be able to provide a thorough investigation of this scientific question. Second, the data for leisure time physical activity and social integration were obtained by self-report questionnaires at one time point. Different participants may have different interpretations of questions. Recording physical and social activity over time would improve computation. Third, we could not repudiate the effect of AD susceptibility genes on low-educated participants, such as apolipoprotein E4 allele, presenilin 1 gene, and amyloid precursor protein gene. Fourth, the cognitive tests used in this study are limited to psychomotor speed, attention and working memory. The other domains of cognitive function such as visuospatial functions, episodic and semantic memory are not included. The associations between GCH metrics and the other cognitive domains are worthy of future research.

## Conclusion

Our study highlighted that a higher score of GCH metrics, indexed by modifiable cardiovascular risk factors, social integration, physical activity, and education, was associated with better cognitive function performance in the young adult population. After controlling for other covariates, formal education and ideal physical activity were the two most significant factors. These findings emphasize the importance of a preventive strategy for modifiable dementia risk factors to enhance cognitive functioning during adulthood.
